# An eponymous history of the anterolateral ligament complex of the knee

**DOI:** 10.1186/s43019-022-00172-0

**Published:** 2022-12-16

**Authors:** Allison M. Morgan, Andrew S. Bi, Daniel J. Kaplan, Michael J. Alaia, Eric J. Strauss, Laith M. Jazrawi

**Affiliations:** NYU Langone Orthopedic Center, 301 E 17th Street, New York, NY 10010 USA

**Keywords:** Anterolateral ligament, Lateral extra-articular tenodesis, Lemaire, MacIntosh, Ellison

## Abstract

**Background:**

Recent interest has surged in the anterolateral ligament (ALL) and complex (ALC) of the knee. Its existence and role in rotary stability of the knee, particularly in the setting of anterior cruciate ligament (ACL) reconstruction, remains a contentious and controversial topic.

**Understanding the ALC:**

We must review our history and recognize the pioneers who pushed our understanding of the ALL forward before it was popularly recognized as a discrete structure. Additionally, given that many eponyms remain in common use related to the ALC, we must standardize our nomenclature to prevent misuse or misunderstanding of terms in the literature. In this review, modern understanding of the anterolateral ligament complex (ALC) is traced to 1829 by exploring eponymous terms first in anatomy and then in surgical technique. Understanding our history and terminology will allow us to better understand the ALC itself.

**Conclusion:**

This review aims to provide historical context, define terminology, and provide insight into the clinical relevance of the ALC.

## Introduction

Throughout its history, medicine, and in particular orthopedic surgery, has used eponymous terms to describe common presentations, often attributed to the first to describe said structure or pathology. Eponyms continue to be frequently used in orthopedic surgery [1, 2], including, but not limited to, describing different types of fractures, diverse pathologic conditions, physical examination maneuvers, surgical approaches, and surgical instruments [3–6]. While the accuracy and validity concerning the usage of such terms have often been questioned [7, 8], eponyms remain a prominent part of daily practice, and thus it is essential to remember from where, when, and whom we obtained these terms.

The anterolateral ligament (ALL) is a portion of the anterolateral ligament complex (ALC), and an anatomic structure that has had a long and controversial history with a recent surge of interest, particularly when considering primary and revision anterior cruciate ligament (ACL) reconstructions [9–16]. ACL failure rates remain unacceptably high, with re-rupture rates from 0% to 14% and clinical failure reported in 2–26%, with increased rates in high-risk patients such as adolescents, pivoting athletes, or those with ligamentous laxity [10, 17]. Of these failures, some are thought to be due to persistent rotational instability due to ALL incompetence. Recently, the role of ALL reconstruction has become a subject of interest in studies such as the STABILITY and SANTI group investigations [18–20]. Even among patients who do not re-rupture, as many as 10% of patients have residual instability or pivot-shift-like symptoms at long-term follow-up [21]. This renewed interest in the anterolateral complex of the knee has grown exponentially, particularly following Claes’s descriptions in the 2010s that popularized the term “anterolateral ligament.” This term was first used by Terry in 1986 (about the capsulosseous layer of the iliotibial band) and further supported by Veira in 2007 and Vincent in 2012 [22–25]. Yet long before this recent rise in popularity and attention, the ALL was known by many other names throughout orthopedic literature. By investigating eponymous terms for the ALL and related to its treatment, the ALL can be better understood. From Paul Segond’s original description in 1879 and Claes’s descriptions in the 2010s to even more recent debate on the ALL’s existence, the ALL and ALC can be considered the new “dark side of the knee” [9, 22, 23, 26]. It is essential to understand from where we have come in order to advance our science and knowledge further regarding this anatomic and clinically relevant structure. Through this review of eponymous terms, we aim to accomplish three goals: first, to compile the history of this structure and pay homage to orthopedic pioneers; second, to clarify commonly used eponymous terms through a direct review of the original literature; and third, to use these historical insights to better understand the anterolateral ligament complex of the knee and its clinical relevance.

## Anatomical eponyms

### Gerdy’s tubercle

**Fig. 1:**
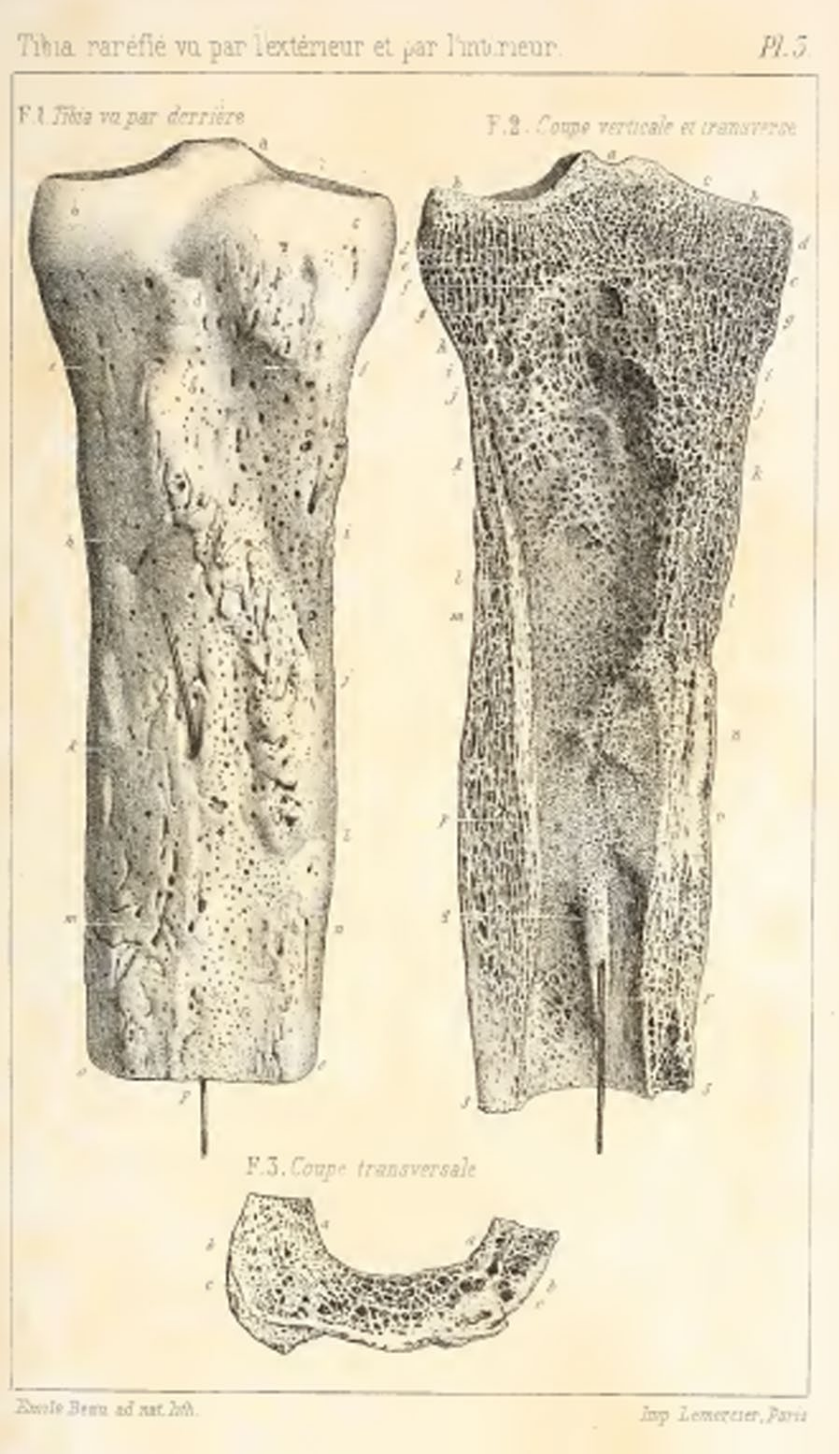
An anatomic illustration of the tibia from one of Gerdy’s textbooks. Reproduced from [29]. Figure out of copyright

Born in 1797, Pierre Nicholas Gerdy began his studies of anatomy at age 16 when he enrolled in a military surgeon program to avoid the general draft in France [27]. His studies were interrupted by the onset of sudden knee arthritis, sending him back home to recover where his local hometown physician gave him his first book on osteology. He recovered and, with this renewed personal interest in bony anatomy, went on to become a professor of surgery and published extensively in surgery and philosophy. Multiple structures have borne Gerdy’s name over the past century, including Gerdy’s ligament, Gerdy’s fontanel, and Gerdy’s fibers; however, these are all now known by other names: the suspensory ligament of the axilla, the sagittal fontanelle, and the superficial transverse metacarpal ligament, respectively [27]. Gerdy’s tubercle was first described in his 1829 text within which he wrote “there exists, at the superior end of the tibia, a tubercle anterior and external which, I believe, escaped the attention of osteologists; the tubercle has important connections to know with the knee joint” (Fig. 1) [28, 29]. Specifically, this anterolateral tubercle is the insertion site of the distal aspect of the IT band. There is little known to explain why this structure of all his accomplishments would become his legacy, but of all the many anatomic landmarks described, his tubercle is the only one still commonly known by Gerdy’s name [27].

### Segond fracture

**Fig. 2:**
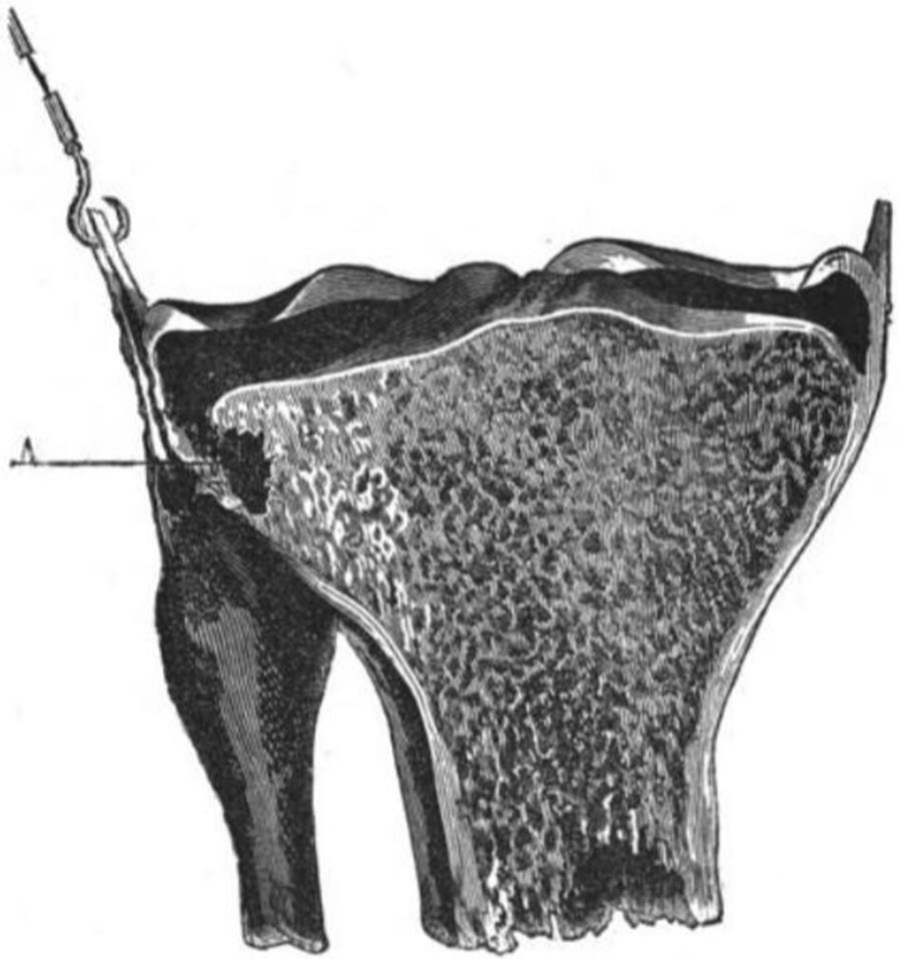
Original illustration of the tibial lesion above and behind Gerdy’s tubercle, the Segond fracture. Reproduced from [26]. Figure out of copyright

Another French surgeon, Paul Segond, trained in general surgery in the nineteenth century and was predominantly interested in urology and gynecology [30–32]. His eponymous fracture came from his only contribution to the orthopedic literature: an 1879 investigation of hemarthrosis in knee ligament sprains [26]. In a cadaveric study, one assistant would steady the cadaver leg while another manually applied force to the limb to simulate various knee injuries. He noted that, in 17/40 cadaver knees to which an internal rotation force to the tibia was applied, there was a “pearly, resistant, fibrous band”—what we now consider to be the ALL—that pulled a portion of cancellous bone off of the anterolateral proximal tibia (Fig. 2). Of note, he described this area as immediately posterior to Gerdy’s tubercle and noted that Gerdy’s tubercle itself never pulled off in this injury pattern [26, 32]. Thus, the first likely descriptions of what is now described as the distal insertion of the ALL were published.

### Kaplan fibers

Born in Ukraine at the turn of the twentieth century, Emanual B. Kaplan’s early medical career was defined by wartime through World War I followed by the Russian civil war [33]. It was in response to the impact of this war that a young Herbert Hoover traveled to Russia on behalf of the American Relief Administration (ARA) and met Dr. Kaplan. The eventual President Hoover was so impressed by Dr. Kaplan and his fluency in five languages that he asked him to begin working for the ARA and encouraged him to emigrate to the USA [33]. In 1924, Dr. Kaplan indeed moved to New York City to become one of the first house staff officers at the Hospital for Joint Diseases, now known as the NYU Langone Orthopedic Hospital. There, and later as a professor of anatomy at Columbia University, he performed countless anatomic dissections and published several texts [33, 34]. Regarding the knee, Kaplan’s now eponymous fibers were described in his 1958 article “The Iliotibial Tract: Clinical and Morphological Significance” in the *Journal of Bone and Joint Surgery*, American Volume [35]. He examined the anatomy of the iliotibial band (ITB) and described fibers connecting the ITB to the femur. These now eponymous Kaplan fibers were recently described in further detail by Herbst et al., described to be distinct fiber bundles, transversely oriented, that connect the superficial ITB to the distal femoral metaphysis. Kaplan fibers insert onto the lateral distal femoral metaphysis as well as via thin accessory fiber bundles proximal and anterior to the lateral femoral condyle. These insertions anchor the ITB and enable its function in rotational control [36].

## Surgical technique eponyms

While a few anatomic eponyms remain in common use for the ALC, countless describe surgical techniques that attempt to reconstruct the disrupted ALC. Andrews, Brückner, Ellison, Hughston, Lemaire, Losee, MacIntosh, Marcacci, Marshall, and Müller procedures represent just a sample of many names associated with published lateral extra-articular tenodesis (LET) techniques [37, 38]. Three eponymous techniques in ALC surgery are reviewed here: Lemaire, Macintosh, and Ellison. Lemaire published the first isolated lateral extra-articular tenodesis in 1967 in France. Around the same time in Canada, Macintosh began working on his techniques, the most popular variation of which he published in the 1970s. In 1979 in the USA, Ellison built off his fellow North American Macintosh’s publications and joined the conversation with his own take on a tenodesis. To review every described LET technique is beyond the scope of this work, but through these three examples we may understand the most influential and persistently cited procedures addressing the ALC.

### Modified Lemaire lateral extra-articular tenodesis

**Fig. 3:**
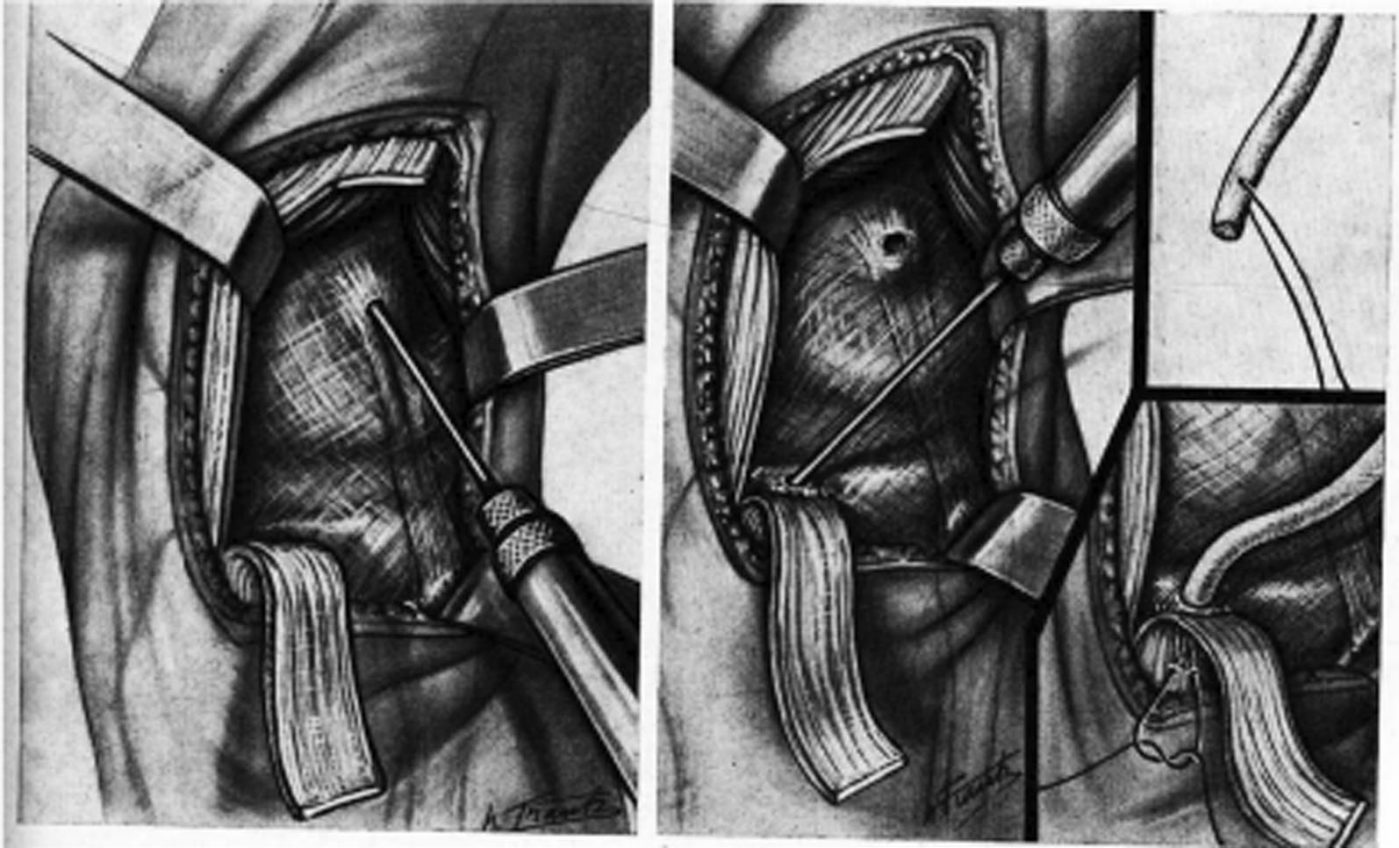
Images of the original Lemaire technique. Reproduced from [40]. Permission obtained from the *British Bone and Joint Journal*

Another French surgeon is remembered in the story of the anterolateral ligament complex through the work of Marcel Lemaire. Little is published regarding his personal history as a general surgeon, but his contribution to the orthopedic realm continues to be valuable. He noted poor results in some of his patients treated for meniscal pathology for whom only the meniscus was addressed, ultimately attributing these poor outcomes to deficiency of the ACL. Thus he began to perform an anterolateral tenodesis to provide stability to the ACL-deficient knee, initially publishing his technique in a series of 46 cases [39]. In his original 1967 technique, an “aponeurotic ribbon” of ITB about 1.5 cm from the posterior margin is dissected and left fixed distally to Gerdy’s tubercle. The ITB is folded down and dissected a few millimeters off the proximal end of Gerdy’s to open up the distal insertion of the graft. A 3 mm bone tunnel is then made just proximal to the insertion of this ITB strip at the proximal end of Gerdy’s tubercle. The LCL is then identified and at the proximal origin, a second 3 mm bone tunnel is created in the lateral femoral epicondyle at the proximal origin of the LCL. Next, the ITB strip is supplemented with “un lacet de nylon,” or a nylon lace to form the “néo-ligament” as Lemaire did not believe the native strip of ITB had enough structural integrity. This “néo-ligament” is formed from the native ITB strip wrapped around the nylon lace and sutured closed. This is then passed into the inferior bone tunnel, tied to the surrounding periosteum and the superior bone tunnel, tensioned, and tied to the surrounding periosteum with thin Nylon sutures (Fig. 3).

Of interest, Lemaire utilizes the eponym Gerdy’s tubercle as well as referring to the ITB as the “*bandelette de Maissiat*,” a still used French eponym not utilized in English [39]. Lemaire would continue to publish on his technique and the good outcomes he found for his patients into the 1970s and 1980s [40, 41]. Christel and Dijan published the first well-known modification in 2002, using a shorter strip of the ITB and twisting it 180° to increase its isometry [42]. Many surgeons have published iterations falling under the umbrella of a modified Lemaire technique since, for example varying in insertion (aiming for isometric or anatomic points), graft length (various lengths shorter than that originally published by Lemaire), and relation of the graft to the LCL [42–46]. The most common variety of the modified Lemaire includes taking a strip of the middle 1/3 of the ITB, leaving its distal attachment to Gerdy’s tubercle intact, and passing the proximal end of the strip deep to the LCL and fixing it proximal and posterior to the LCL origin, either in a bone tunnel with an interference screw or with a suture anchor [47].

### MacIntosh lateral extra-articular tenodesis

While Marcel Lemaire operated in France, across the Atlantic David L. MacIntosh was simultaneously working towards developing his lateral tenodesis technique in Toronto, Canada. As the orthopedic surgeon for University of Toronto in the 1950s, he noted instability contributing to poor outcomes in some of his varsity athletes [48]. He and his colleague Galway described the physical examination maneuver now widely known as the pivot shift test in a 1972 Canadian Orthopedic Association meeting [49]. They would go on to publish a thorough description of the pivot shift in 1980, describing it as “both a clinical phenomenon that gives rise to the complaint of giving way and a physical sign that can be elicited upon examination of the injured knee” [50].

**Fig. 4:**
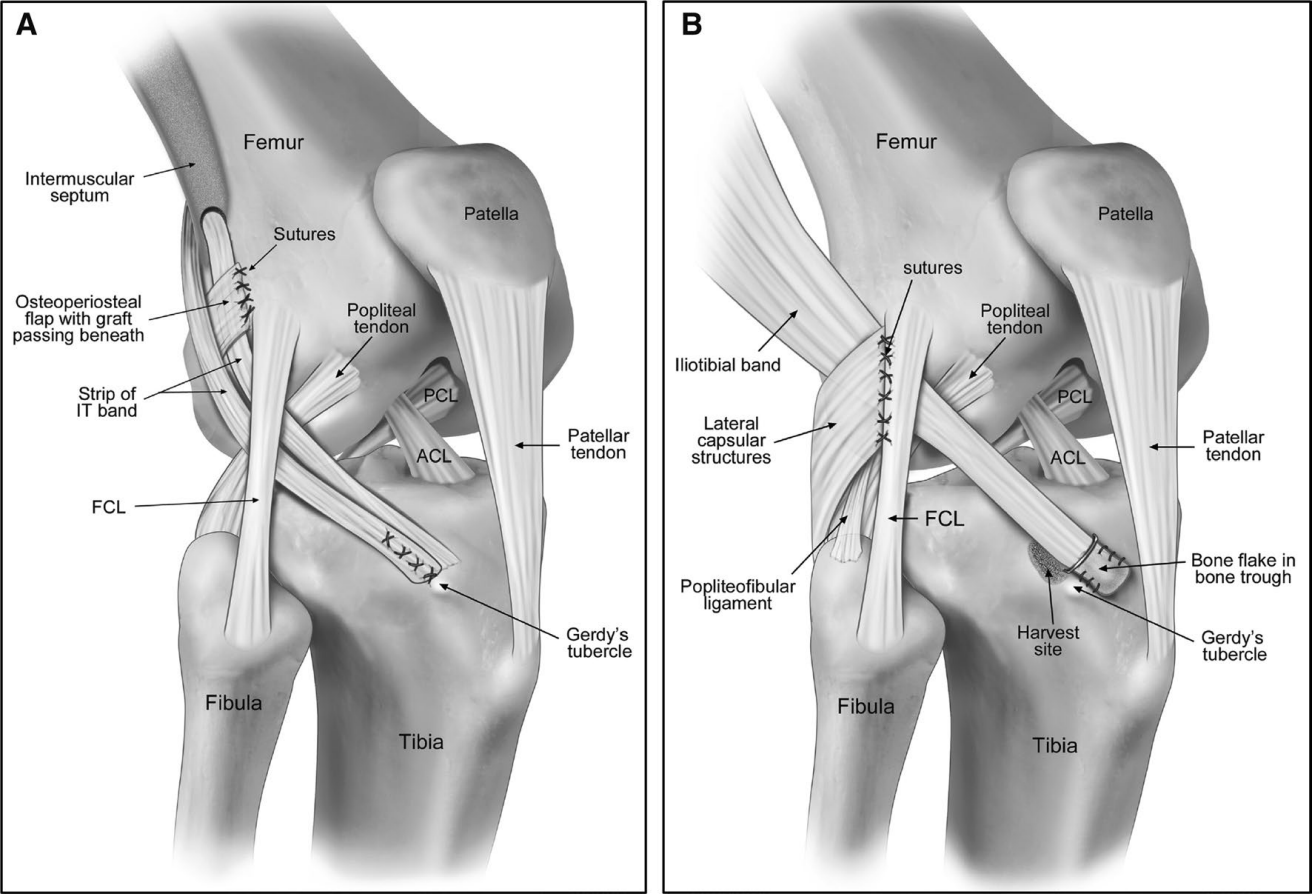
The MacIntosh (**A**) and Ellison (**B**) procedures demonstrating the primarily soft tissue fixation and lack of intra-osseous bone tunnels in the MacIntosh and the distal harvest of the iliotibial band in the Ellison. Reproduced from [38]. Permission obtained from Elsevier

In his treatment of these patients with instability, MacIntosh would go on to develop his eponymous soft-tissue LET technique. In his method, a strip of the middle third of the ITB is dissected, leaving it attached distally to Gerdy’s. This graft is then passed retrograde under the LCL, through a soft-tissue subperiosteal tunnel posterior to the femoral origin of the LCL, through the lateral intramuscular septum and turned distal, passed back under the LCL, and sutured back onto itself at Gerdy’s tubercle (Fig. 4A) [51, 52]. This was a unique technique as it involved primarily soft tissue fixation without the use of intraosseous tunnels or bony fixation. Tracing the refinement of his techniques, his 1960s entirely extraarticular procedure is sometimes referred to as MacIntosh I while his 1970s iteration, including an intraarticular portion, is known as MacIntosh II and is most similar to the current use of the eponym. Finally, in the late 1970s or 1980s, he developed the MacIntosh III in which the patellar tendon was used to augment the acutely repaired ACL. Given that this technique involved ACL repair rather than reconstruction, the MacIntosh III has fallen out of favor, though as with most trends in orthopedics, we may see a similar technique develop as the popularity of ACL repair increases [48]. As of the time of this publication, the term MacIntosh technique most commonly refers to a MacIntosh II-type procedure. After his retirement in 1984, the University of Toronto renamed the Hart House clinic the David L. MacIntosh Sports Medicine Clinic in honor of his many achievements.

### Ellison lateral extra-articular tenodesis

An American surgeon and longtime resident of New England, Arthur E. Ellison was passionate about sports surgery with a particular interest in skiing [53]. He served as a team physician for the US Ski Team as well as the Williams College football team [54]. Throughout these pursuits, he, like the authors described before him, became interested in knee instability experienced by the variety of athletes he cared for ranging from football, basketball, skiing, hockey, soccer, and even snowmobile. In his eponymous LET technique, the distal insertion of the ITB is released along with a 1.5 cm piece of bone. The ITB is then dissected proximally and then passed deep to the LCL and lateral capsule, but superficial to the popliteus tendon. The bone block is then fixed at its new insertion in a bone trough, just anterior to Gerdy’s tubercle, and fixed with a staple and sutured to the lateral fibers of the patella tendon. The lateral capsule is then imbricated to the proximal posterior edge of the LCL. Now traveling beneath the LCL, the ITB is tensioned by the LCL in movement to dynamically stabilize the tibia and prevent anterior translation relative to the femur as the knee extends (Fig. 4B) [55].

In 1979, Ellison published the results of this procedure on 18 patients with good results and return to sport, concluding that “stability can be restored by retaining the lateral tibial condyle posteriorly by transplanting the iliotibial band posteriorly, in a flexor position, thus preventing it from going into an anterior or subluxing position” [55]. Standing out from the multitude of other LET procedures due to the distal release of ITB, the Ellison procedure canonizes the name of another great sports surgeon in the world of the ALC.

## Conclusion

The purpose of this review was threefold. First, we aimed to compile the history of pioneers in orthopedic surgery and discoveries surrounding the ALC of the knee into one manuscript. The contributions to anatomy made by Gerdy, Segond, and Kaplan and to surgical techniques by Lemaire, Macintosh, and Ellison should be remembered. Second, we aimed clarify and define these commonly used eponyms to ensure our use of eponyms is accurate and consistent. Eponyms can be useful in areas such as ALC surgical techniques, where each name serves as a single distinct identifier for a specific technique. However, they also pose challenges, as the plethora of names and minor nuances between procedures can be lost and, when the initial surgeons’ works are not reviewed and remembered, their names can be easily misused. Herein, the original texts leading to eponyms were reviewed to assure the most accurate and precise terminology. Third, with controversy regarding the ALC and ALL, history has the unique potential to provide insights into where we have obtained our current foundation of knowledge. By investigating our history closely and standardizing the nomenclature surrounding the ALC, we can forge ahead to a greater understanding of this controversial and clinically relevant area of the knee.

## Data Availability

Not applicable.
